# Personality traits and psychiatric comorbidities in alcohol dependence

**DOI:** 10.1590/1414-431X20155036

**Published:** 2015-11-27

**Authors:** M.F. Donadon, F.L. Osório

**Affiliations:** 1 Departamento de Neurociência e Comportamento, Faculdade de Medicina de Ribeirão Preto, Universidade de São Paulo, Ribeirão Preto, SP, Brasil; 2 Instituto de Tecnologia para Medicina Translacional, Ribeirão Preto, SP, Brasil

**Keywords:** Alcoholism, Dependence, Personality traits, Comorbidity

## Abstract

Non-adaptive personality traits may constitute risk factors for development of psychiatric disorders such as depression and anxiety. We aim to evaluate associations and the predictive value of personality traits among alcohol-dependent individuals, with or without psychiatric comorbidities. The convenience sample comprised two groups of males over 18 years of age: one with subjects who had an alcohol dependence diagnosis (AG, n=110), and a control group without abuse and/or alcohol dependence diagnosis (CG, n=110). The groups were assessed by means of the Structured Clinical Interview DSM-IV (SCID-IV). AG participants were recruited among outpatients from the university hospital, whereas CG participants were recruited from a primary healthcare program. Data collection was done individually with self-assessment instruments. Parametric statistics were performed, and a significance level of P=0.05 was adopted. A positive correlation was observed between openness and the length of time that alcohol has been consumed, as were significant and negative correlations between conscientiousness and both the length of time alcohol has been consumed and the number of doses. For alcoholics, extraversion emerged as a protective factor against depression development (P=0.008) and tobacco abuse (P=0.007), whereas openness worked as a protective factor against anxiety (P=0.02). The findings point to specific deficits presented by alcoholics in relation to personality traits with or without psychiatric comorbidities and to the understanding that therapeutic approaches should favor procedures and/or preventive measures that allow more refined awareness about the disorder.

## Introduction

Epidemiological studies of alcoholism reveal some interesting insights. Approximately two billion people use alcohol worldwide, and 76.3 million of them have at least one disorder caused by their alcohol use (1). Statistics show that impacts from the alcohol consumption habit are extremely negative and associated with a series of detriments. Clinically, one can highlight hepatic cirrhosis, neoplasia, gastritis, oesophageal varices, pancreatitis, and diabetes mellitus, among others. At the psychiatric level, the literature shows an elevated percentage of mood and/or anxiety disorder comorbidities, a fact associated with worse prognoses and to difficulty in adhering to treatment (2 3 4).

The literature has shown multiple variables that predispose alcoholism, highlighting genetic aspects (5,6), gender (7), age (1), social influence (8) and personality factors (9,10), among others.

Studies on personality traits reveal a special relationship with alcoholism. Such traits can be defined as a set of qualitatively measured features that allow for assessment of specific individual disparities (11). An empirical and comprehensive study by Digman suggested a personality model comprising five factors. His study was based on the work of other researchers such as Tupes, Cristal and Cattell, and these authors were further referenced by Costa and McCrae (12).

According to the model, there are five factors or personality traits: *a*) neuroticism (which is linked to anxiety, anger, impulsiveness, negative affect and psychic suffering); *b*) extraversion (which is reinforced by positive emotions and linked to the individuals' degree of sociability, assertiveness and communication); *c*) openness (which is connected to curiosity, beliefs, flexibility, independent judgment, taste for complexity and novelty, as well as to non-conventional experiences); *d*) agreeableness (which refers to altruistic traces and is linked to sympathy and cooperation tendencies); and *e*) conscientiousness (which is linked to self-control, planning, discipline, determination and organization) (12,13).

Previous clinical studies indicate that neuroticism features and diminished consciousness traits are risk factors for alcoholism development. At the same time, the association of alcoholism with traits such as openness, kindness and extraversion was not considered to be meaningful (14,15).

Besides such associations, research has shown an important link between the amount of alcohol consumed and personality traits. A study involving university students who presented a high alcohol consumption pattern indicated that consumption reduction is significantly connected to high awareness and low neuroticism indicators (16).

Some authors also point out the important associations between personality traits and the presence or absence of psychiatric disorders. Alcoholics who present depressive disorder have more expressive neuroticism indicators and less awareness in comparison to those without depression (17,18).

The current study addresses two issues: the importance of new research to help better understand personality as an alcoholism predictor and the lack (to our knowledge) of studies conducted with Brazilian samples. The present paper aims to assess personality trait associations and their predictive value for alcohol dependence by highlighting the presence or absence of other psychiatric disorders.

## Material and Methods

### Participants

The current study used a convenience sample of males over 18 years of age. Two groups of 110 subjects each were constructed. Group AG individuals were recruited from the alcoholic hepatic disease ambulatory treatment facility at Hospital das Clínicas, Faculdade de Medicina de Ribeirão Preto, Brazil. All AG subjects were diagnosed with alcohol dependence according to criteria from the Diagnostic and Statistical Manual of Mental Disorders - DSM 4th edition (DSM-IV). Group CG subjects had no alcohol abuse or dependence diagnosis and were recruited from primary healthcare services (linked to the Faculdade de Medicina de Ribeirão Preto, Brazil) and from a non-governmental organization with social objectives and training in general services (for example: animal care, manicure). Recruitment sites were chosen to balance the economic characteristics of the two groups. The groups were also matched by gender, age, and education.

### Instruments

Several instruments were used in the assessment: *a*) the Structured Clinical Interview from DSM-IV (SCID-IV - clinical version), suggested by First et al. (19) and translated and adapted to Portuguese by Del-Ben et al. (20). Only module E was used in the current study in order to carryout an alcohol dependence diagnostic investigation. *b*) Five Personality Factors Inventory - NEO Revised (NEO-FFI-R - short version): an instrument developed by Costa Jr and McCrae (21). It comprised 60 items scored on a Likert scale from 0 to 4 points. The scale gives personality feature indicators based on the five-factor theory. The instrument was validated and translated to Portuguese by Flores-Mendoza (22). *c*) Beck Anxiety Inventory (BAI): a 21-item instrument that assesses the presence and intensity of anxiety symptoms. The version by Cunha (23) was translated and adapted to a Brazilian Portuguese version and was used in the present study. Twenty was set as the cut-off score to indicate possible anxiety pathologies. *d*) Patients' Health Quality - 9 (PHQ-9): a 9-item instrument for rating the presence of depressive symptoms. The Brazilian Portuguese version validated by de Lima Osório et al. (24) was used. Ten was set as the cut-off score to indicate a possible depressive condition. *e*) Fagerström's Nicotine Dependence (FTND): a 6-item measure of nicotine physical dependence level. The version by Carmo and Pueyo (25) translated and validated to Brazilian Portuguese was used. Seven was set as the cut-off score for nicotine dependence. *f*) Socio-demographic and Clinical Questionnaire: an 18-item instrument exclusively developed for the present study and used to collect complementary data for socio-demographic and clinical identification. All instruments except *a*) and *f*) were self-administered.

### Procedure

*Data collection*. First, two copies of the information and consent form were given to those who agreed to participate. After signing them, the participant would keep one copy and the other would be filled out according to the data collection protocol. Next, module E of SCID-IV was conducted to confirm or rule out alcohol dependence. Subsequently, a packet containing the Socio-demographic and Clinical Questionnaire, BAI, PHQ-9, FTND and the NEO-FFI-R was handed out.

The completion of the self-administered instruments was done in front of a researcher, who was available to answer any questions. Data were collected individually and transcribed into a database.

### Data analysis

Data were analyzed by *a*) descriptive statistics: socio-demographic and clinical features analyses, *b*) parametric statistics: Student's *t*-test (group comparisons), Pearson's correlation test (variable correlations), and multivariate logistic regression (predictive variable analysis), using the Statistical Software Package for Social Sciences (SPSS, version 15, USA). The adopted significance level was P<0.05.

### Ethical considerations

The study met the ethical parameters for research with human beings and was approved by the Local Ethics Committee at Hospital das Clínicas da Faculdade de Medicina de Ribeirão Preto, Brazil (Process 005/2012).

## Results

The main clinical and socio-demographic data are reported in Table 1. Most participants were married, mean age was approximately 53 years, and education levels were mainly at the elementary and high school levels. No significant differences were found for these variables between the two groups. However, a significantly higher percentage of the CG was professionally active compared to the AG group. AG presented higher nicotine dependence levels - a depression and anxiety indicator - and this difference was statistically significant compared to CG.

**Table:**
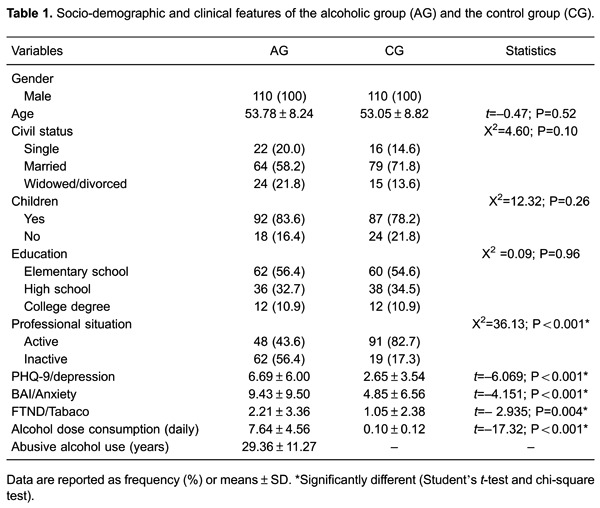

Table 2 highlights the statistically significant differences between AG and CG for all of the five assessed personality factors; all factors were less evident in AG.

**Table:**
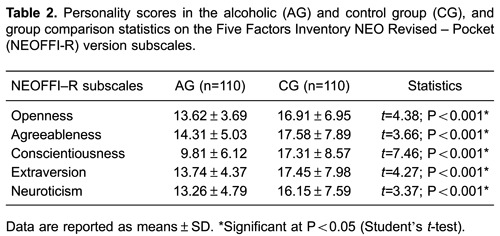

In order to highlight the possible relationship between alcohol dependence and other personality traits, we also correlated traits with alcohol dose and the length of time that alcohol has been consumed among AG participants. There was a positive correlation between the openness factor and the length of alcohol consumption (r*=*0.14; P=0.05). In contrast, a negative correlation was observed between the conscientiousness factor and length of consumption (r=-0.18; P=0.008). Similar findings emerged when number of doses consumed was substituted for length of alcohol consumption (r=-0.15; P=0.03). All correlations were of low magnitude. Non-significant correlations were observed for the other traits.

A multivariate logistic regression was carried out to evaluate the value of personality traits for predicting alcohol dependence. This regression took all five factors under consideration. As Table 3indicates, extraversion was the only statistically significant predictor. Each increase of punctuation in this factor decreased the chance of the disorder worsening by 19%.

**Table:**
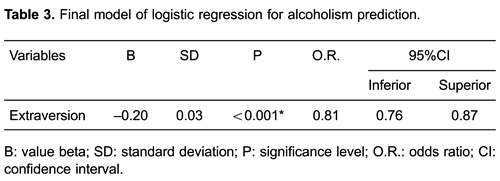

New regression analyses were performed to assess the predictive value of different personality traits in the development of different psychiatric disorders among alcoholics. AG was subdivided according to the presence or absence of such disorders based on the cut-off score, which was standardized through specific instruments. Thirty-six subjects (32.7%) presented depression indicators, based on the BAI scale; 13 (11.8%) presented moderate anxiety indicators, based on the BAI scale; and 18 (16.4%) presented nicotine abuse disorder, based on the FTND scale.

Table 4 presents the different personality traits within these groups. Conscientiousness traits prevailed among alcoholics who presented depressive disorder. Extraversion traits were more evident among alcoholics who did not present depressive disorder or nicotine abuse disorder. No significant differences were found in the other personality traits and disorders.

**Table:**
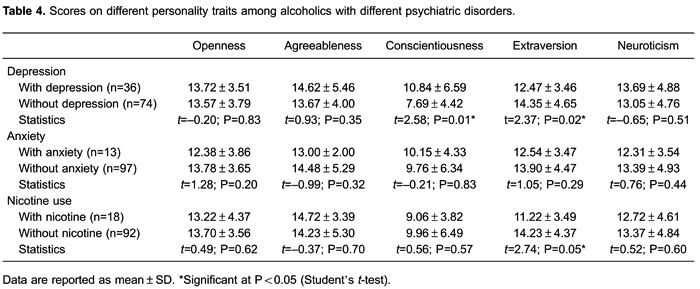

Based on the findings, regression analyses were performed to establish the predictive value of those variables. A multivariate model was tested and confirmed the presence of each of those disorders. The variables with a P<0.20 tested in the initial design were introduced in the group comparison analysis.

Extraversion emerged as a negative predictor of depression (Odds=0.91; 95%CI=0.84-0.97; P=0.008) and smoking (Odds=0.77; 95%CI=0.64-0.93; P=0.007) in AG. The openness factor was also associated with a lower likelihood of anxiety disorder in this group (Odds=0.85; 95%CI=0.74-0.98; P=0.02).

## Discussion

The present study assessed the possible correlation between personality traits and different troubling aspects of alcoholism. Such correlations may be viewed as risk or protective factors against the evolution of these difficulties.

All the factors were less frequent in the alcoholic group, and this finding is consistent with data in the literature (14,26,27), which has documented the lower presence of agreeableness, openness and extraversion among alcoholics; it is especially consistent with the meta-analysis by Malouff et al. (28).

Extraversion emerged as a negative predictor of alcohol dependence and disorders such as depression and smoking. A possible hypothesis to justify this finding is that individuals with lower indicators of such a trait are more introspective or shy, which may lead to high-risk behaviors such as the abuse of alcohol and other substances as a confrontation strategy (29).

However, these results contrast with the previous literature, which points out that increased alcohol consumption is associated with increased extraversion traits (30). A possible explanation for such conflicting findings would be that these studies used a descriptive methodology, and lacked a comparison group of healthy participants, such as the one used in the present study. Additionally, these studies used samples of both genders. Previous studies involving the general population showed that women present stronger extraversion traits than do men (31). The sample used in the present study was exclusively male, so, the present research has allowed a detailed look at the influence of this factor on the male gender.

The openness trait emerged as protective factor against anxiety among alcoholic subjects. High scores in this personality trait indicate individuals who are curious, sensitive and flexible. Those who readily seek to engage in new experiences may experience a more conscious sense of control, which may result in reduced perceptions and experiences of fear, tension and consequently anxiety (32). However, openness traits, i.e., a stronger tendency to question rules and patterns and to seek non-conventional experiences, are associated with alcohol chronicity, which leads to early and constant use (33)

Previous studies (27) indicate that low conscientiousness trait indicators may be associated with high alcohol consumption as well as high relapse rates, since low conscientiousness favors little effort to change one's own behavior due to lack of discipline, planning, self-control and persistence. According to the present study, this personality trait is inversely associated with the length of time alcohol has been consumed and the number of consumed doses. This is consistent with previous findings by Ruiz et al. (26).

The present study highlights the stronger presence of this personality trait among subjects with depressive disorder. The data extend the work of Krueger et al. (17), which showed that high level of self-control, discipline and planning are correlated to depression development among alcoholic subjects. This may be due to increased guilty feelings because of unaccomplished duties and/or goals that were not reached.

Agreeableness, according to previous literature (28), was also lower among alcoholic subjects. It suggests that less altruistic and more egocentric individuals, with low cooperation tendency, are more apt to be abusive and to develop alcohol dependence.

As for the neuroticism factor, the findings were opposite to those in previous studies, which would lead us to expect the presence of impulsivity, anger and negative emotions in alcoholic subjects. A possible explanation is that a social desirability bias occurred in the current study; i.e., because self-assessment instruments were used, subjects' responses may not have reflected their actual perceptions and experiences.

Another explanation relates to characteristics of the sample. Previous studies have flagged neuroticism traits as stronger in women than in men (31,34). The fact that our sample had only male subjects may have once again diminished the influence of this variable. We can only speculate on the influence of low level personality traits as risk factors to alcoholism.

Finally, it is possible to conclude that personality traits play an important role in the development of addictive behavior such as alcohol dependence and associated disorders. That is why they are important factors to be considered during interventions that aim to diminish and/or treat this clinical condition and its impact and/or severity. However, some traits may also afford protection, for example, from risk behaviors such as early and abusive alcohol use, which would in turn reduce the likelihood of developing alcohol dependence.

The present study has some limitations: *a*) it used screening tools to assess clinical comorbidities like depression, anxiety and nicotine dependence rather than instruments taken as the gold standard for this kind of diagnostic like SCID; *b*) it lacked systematic scales to evaluate the severity of alcoholism.
